# Anorectal Injuries due to Foreign Bodies: Case Reports and Review of the Management Options

**DOI:** 10.1155/2013/809592

**Published:** 2013-03-03

**Authors:** Faruk Karateke, Koray Das, Sefa Ozyazici, Ebru Menekse, Zikret Koseoglu, Ozgur Karcioglu

**Affiliations:** ^1^Department of General Surgery, Numune Training and Research Hospital, 01170 Adana, Turkey; ^2^Department of Emergency, Numune Training and Research Hospital, 01170 Adana, Turkey; ^3^Department of Emergency Medicine, School of Medicine, Acıbadem University, Bakırkoy, 34140 Istanbul, Turkey

## Abstract

Anorectal injuries due to autoerotic activity with rectal foreign bodies were identified in four male patients. The objects were bottle in one patient, glasses in two patients, and showerhead in one patient. Foreign bodies were extracted within lithotomy position after anal dilatation, under general anesthesia in 3 patients. One patient presented with peritoneal irritation and had a diagnosis of rectal perforation. He underwent transanal rectal repair with proximal fecal diversion. In this paper we described 4 patients who had anorectal injuries due to autoerotic activity with foreign bodies and reviewed the management options in literature.

## 1. Introduction

Rectal foreign bodies (RFBs) inserted in the rectum and their management have been reported in the literature with dating back to 16th century [1, 2]. RFBs are settled in the rectum via either of two ways: those inserted per annum and more rarely ingested by the mouth [3]. The oral way is the case mostly encountered in those with poor intellect, mentally retarded, and senile or debilitated persons, also in drug trafficking. On the other hand, RFBs inserted in the rectum per anally are noted most commonly in middle-aged men in context of autoerotic instrumentation [4, 5]. In this paper we described four patients admitted to the emergency department (ED) with RFB related autoerotic activity and reviewed management options in the literature.

## 2. Case Presentations

Patients admitted with anorectal injuries related autoerotic activity were enrolled for the study. Written consent has been obtained from all patients to be included in our paper. Characteristics of the patients were presented in Table 1.

## 3. Case 1

**Figure 1:**
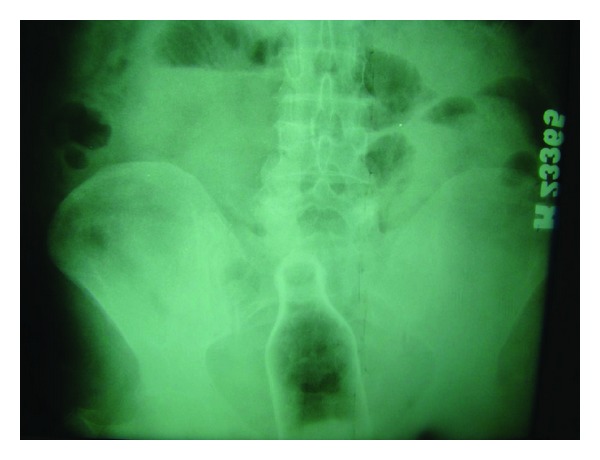
X-rays revealed a bottle visualized in the rectosigmoid area.

Sixty-six years aged male patient was admitted to ED. The patient acknowledged that he was drunk and inserted the bottle in rectum for erotic activity. The bootle was palpated in 5-6 cm distance in digital rectal examination. Plain abdominal X-rays revealed an image of bottle visualized in the rectosigmoid area, without associated free air (Figure 1). After failed attempts in the ED, the patient was transferred to the operating room (OR). The bottle was extracted manually in the OR in lithotomy position under general anesthesia (GA) following anal dilation. Superficial mucosal lacerations are visualized in rigid rectosigmoidoscopy (RSS). The patient was discharged after an uneventful course in the hospital at 24 hours.

## 4. Case 2

**Figure 2:**
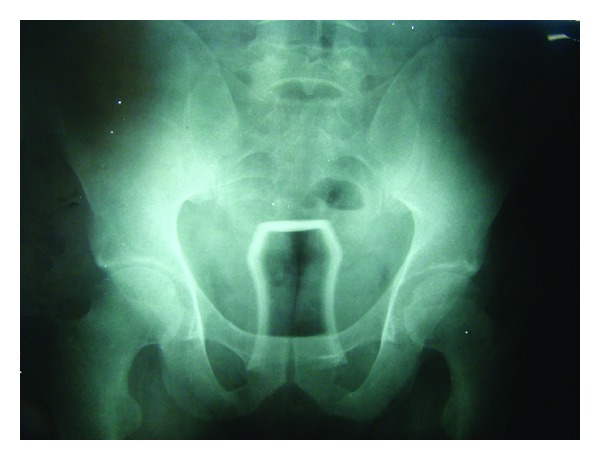
X-rays showed a glass inserted in the rectum.

A middle-aged male patient was admitted to the ED due to pain in the groins and rectal bleeding. He reported to have unusual sexual habits. He inserted glass of tea in rectum and thereafter tried to take it out himself but he gave up when noticed anal bleeding (Figure 2). He was administered broad spectrum antibiotics and tetanus prophylaxis. Anorectal examination revealed lacerations around anal mucosa with internal sphincter injury. The patient was taken to the OR and the glass was extracted manually under GA. The patient was discharged without sequelae in 24 hours.

## 5. Case 3

A young man with no remarkable medical history was admitted with severe abdominal pain, nausea, bloody diarrhea, and fever which had started two hours ago. It was noticed that he had inserted the showerhead to his anus for sexual arousal and afterwards tried to extract himself. On physical examination he had acute abdomen with a fever of 39°C. Plain X-rays failed to visualize any abnormal image, including free air. Abdominal ultrasound disclosed free fluid between bowel segments. The patient was transferred to the OR to perform RSS under GA. Following detection of full-thickness rectal injury and perforation of about a radius of 3 to 4 cm in the anterior wall, the defect was sutured primarily and repaired. Loop colostomy was performed for diversion because of fecal contamination. He was discharged in the 7th day without sequelae after scheduled for colostomy closure after 3 months.

## 6. Case 4

**Figure 3:**
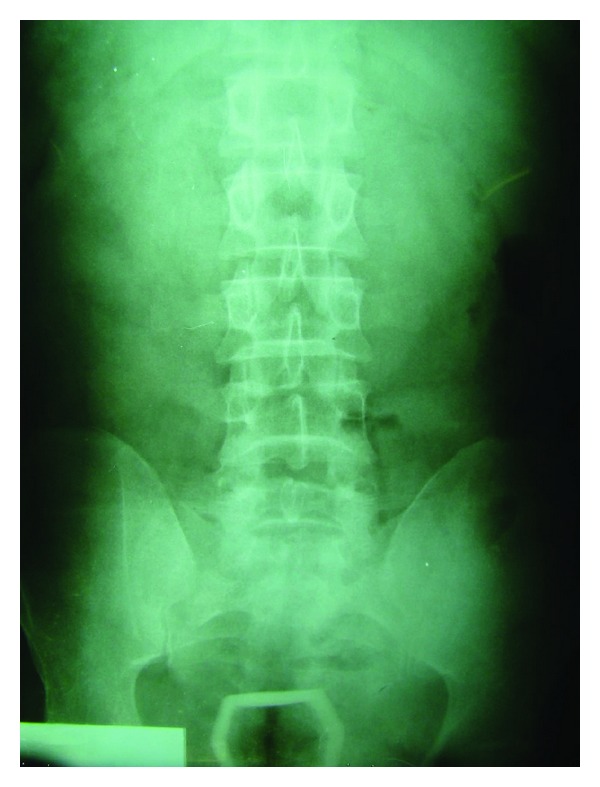
X-rays visualized a glass leading obstruction.

A middle-aged man was referred to the ED with severe anal pain and bowel obstruction. In medical history he described to use glass as a sex toy. He failed to extract it himself. Digital rectal examination revealed the RFB in 5 to 6 cm distance to the anal verge. X-rays revealed no finding consistent with visceral perforation and the opacity of the glass was visualized in the rectum (Figure 3). Following unsuccessful extraction attempts in the ED, the patient was taken to the OR. After anal dilation in lithotomy position the object was extracted using forceps and Allis penses under GA. Postextraction rigid RSS revealed superficial mucosal lacerations in rectal walls. The patient was discharged after an uneventful course in 24 hours with normal stool habits.

## 7. Discussion

Anorectal foreign bodies can either be ingested orally or inserted anally. The vast majority are inserted for autoerotic purposes, and the majority of these patients are middle-aged homosexual men [4–8]. There are a myriad of different kinds of RFB described in the literature. These objects include bottles, glasses, cans, jars, umbrellas, vegetables, and stones in different sizes and shapes [3]. More rarely, some drugs used to treat itching, constipation, hemorrhoids, or rectal prolapse can be inserted into rectum and stayed there [3]. Whether done for purposes of sexual enjoyment or not, voluntarily or accidentally, the reported incidence of RFB has been increasing [7, 8]. It still remains an important problem for ED physicians and general surgeons in their approach with a variety of management options of anorectal injuries resulting from the insertion or extraction of the RFB.

Depending on the surrounding cultural and social milieu, patients mostly delay referral to hospital, fabricate some fake stories, and hide the thorough history [9]. Abdominal or pelvic pain, obstipation, tenesmus, and rectal bleeding are the most commonly recorded complaints on admission in the ED [3]. Patients frequently try to take the objects out themselves in order to disguise the event from the public. These maneuvers may cause the objects to displace more proximally and lead to rectal injury [3, 10]. A careful physical examination should be performed to determine sphincter competency and also workup including direct X-rays or abdominopelvic series of computed tomography in the suspicion of perforation. RSS should be performed for an appropriate diagnosis, and a genitourinary trauma must be rule out.

Various work up and management algorithms for patients with rectal foreign bodies have been described in the literature. After a complete assessment, manual extraction attempts transanally is suggested as an initial treatment of choice in patients without signs of perforation. This maneuver is successful in the majority of cases [7, 11]. It can be performed under pudendal nerve block and spinal anesthetic and/or intravenous sedation as needed to help the patient relax, decrease anal sphincter spasm, and improve exposure. If the foreign body is located high in the rectum or even in colon endoscopic approach may be helpful in cases and a long Kocher clamp or ringed forceps can be used for extraction. Lake et al. [8] reported that when the RFB was in the sigmoid approximately 55% of cases eventually required laparotomy for removal, as opposed to only 24% in cases of rectal objects. If transanal and endoscopic approaches fail to extract the foreign object or there are peritoneal signs, the patient needs to be taken for surgery. Laparoscopic attempt have been recommended to push the RFB distally to allow for transanal removal by some authors [12, 13]. Laparotomy is required in the case of laparoscopic failure or gross fecal contamination. If the objects are inadequate, a colotomy can be performed. Lacerations of the colon involving less than one third to half the circumference and are fresh or without gross peritoneal contamination can be repaired primarily. Diversion should be performed in patients with delayed presentation, gross fecal contamination, signs of abdominal sepsis, or hemodynamic instability.

Postextraction rectal injury should be evaluated immediately and thoroughly with RSS and the patients must be followed up in the hospital at least 24 hours after the procedure. RFB may cause long-term complications including rectal inflammation, perforation and resultant peritonitis, perirectal abscess, and fistulae. If there is evidence of sphincteric injury, surgical repair should be delayed [7, 8].

## 8. Conclusion

Autoerotic activity with rectal foreign bodies may cause life-threating rectal injuries including lacerations, bleeding, perforation, and obstruction. An orderly approach is essential for the diagnosis, management, and postextraction evaluation of the patient with a rectal foreign body.

## Figures and Tables

**Table 1:** Characteristics of patients with anorectal injuries related to autoerotic activity.

*N*	Age	Sex	RFB	Injury	Management/procedure
1	66	M	Bottle	Rectal lacerations	GA/manual extraction in OR
2	36	M	Glass	Bleeding, sphincter injury	GA/manual extraction in OR
3	20	M	Showerhead	Bleeding, perforation	GA/Diversion colostomy
4	55	M	Glass	Rectal obstruction	GA/forceps extraction in OR
